# Perspectives of Phage Therapy in Non-bacterial Infections

**DOI:** 10.3389/fmicb.2018.03306

**Published:** 2019-01-09

**Authors:** Andrzej Górski, Paul L. Bollyky, Maciej Przybylski, Jan Borysowski, Ryszard Międzybrodzki, Ewa Jończyk-Matysiak, Beata Weber-Dąbrowska

**Affiliations:** ^1^Ludwik Hirszfeld Institute of Immunology and Experimental Therapy, Polish Academy of Sciences, Wrocław, Poland; ^2^Department of Clinical Immunology, Medical University of Warsaw, Warsaw, Poland; ^3^Division of Infectious Diseases, Department of Medicine, Stanford University Medical School, Stanford, CA, United States; ^4^Immunology Program, Stanford University, Stanford, CA, United States; ^5^Department of Medical Microbiology, Medical University of Warsaw, Warsaw, Poland

**Keywords:** phage, phage therapy, viral infection, fungal infection, drug repurposing

## Abstract

While the true value of phage therapy (PT) in human bacterial infections still awaits formal confirmation by clinical trials, new data have been accumulating indicating that in the future PT may be applied in the treatment of non-bacterial infections. Thus, “phage guests” may interact with eukaryotic cells and such interactions with cells of the immune system may protect human health (Guglielmi, 2017) and cause clinically useful immunomodulatory and anti-inflammatory effects when administered for therapeutic purposes (Górski et al., 2017; Van Belleghem et al., 2017). Recently, a vision of how these effects could translate into advances in novel means of therapy in a variety of human pathologies secondary to immune disturbances and allergy was presented (Górski et al., 2018a). In this article we present what is currently known about anti-microbial effects of phage which are not directly related to their antibacterial action and how these findings could be applied in the future in treatment of viral and fungal infections.

## Introduction

The growing threat of antibiotic resistance has contributed to a rapidly increasing interest in phage therapy. Recently, this subject was discussed in detail; in fact, reviews on phage therapy appear almost every month (Górski et al., 2018c). The first clinical trial of PT performed in accord with Good Medicinal Practice and evidence-based medicine showed a clinically relevant reduction in bacterial burden after 7 days of topical phage application (Jault et al., 2018). Hopefully, this and other planned and ongoing trials will lead to the licensing of phage products as anti-infective agents (Sansom, 2015).

Recent progress in studies on phage has provided new insights into the biology of bacterial viruses. Phage may mediate anti-inflammatory and immunomodulating activities that could be relevant in the maintenance of immunological homeostasis (Górski et al., 2017). Thus, phage present in our body (a phageome) could protect human health (Guglielmi, 2017). Furthermore, those studies also suggest the potential of PT in autoimmune diseases and allergy (Górski et al., 2018c).

Could our phageome and PT also protect us not only from bacterial but also from at least some viral and fungal infections? The treatment of viral infections remains a difficult challenge, whilst the development of biologic therapies has increased with the concurrent increase of their substantial side risk of viral infections (Noreña et al., 2018). As pointed out, most viral infections lack specific treatment and the need to combat old, emergent and re-emergent viruses is not paralleled by the development of new antivirals (Mercorelli et al., 2018). Likewise, invasive fungal infections have been increasing while current antifungal agents have important limitations such as their limited choice, severe toxicity, high cost and emergence of drug resistance (Casadevall, 2018; Li et al., 2018; Zheng et al., 2018). Therefore, alternative and safe anti-fungal agents are also urgently needed.

Drug repurposing, the application of an existing therapeutic to a new disease indication is currently a popular strategy offering the hope for rapid clinical development with fewer risks and a lower cost than *de novo* drug development (Corsello et al., 2017). Metformin is the first-line treatment for type 2 diabetes that suppresses gluconeogenesis (Madiraju et al., 2014). Recent data indicate that this globally most prescribed antidiabetic medication also functions at the level of the microbiome specifically reducing the abundance of *Bacteroides fragilis* in the intestines, which increases the level of liver bile acids and eventually increases insulin sensitivity. Moreover, it has also been shown to have anti-cancer and longevity promoting properties (Guo and Xie, 2018). The drug repurposing strategy has already resulted in identification of new an antiviral agents (e.g., quinine as antiviral against dengue virus infection) (Malakar et al., 2018). Further studies are warranted to confirm that this strategy applied as PT repurposing might be successful in the treatment of some viral and fungal infections.

There were observations on antiviral activity of phage in the 1960s and 70s (Międzybrodzki et al., 2005). However, it has to be kept in mind that those results were achieved using non-purified phage lysates. Therefore, it cannot be excluded that the observed effects were caused by bacterial remains rather than phage themselves.

It was demonstrated that *Escherichia coli* K12 phage was active against herpes simplex virus (HSV) and vaccinia virus *in vitro* (plaque inhibition assay on chick embryo monolayer cultures) and *in vivo* (a herpetic corneal ulcer model in rabbit). An antiviral agent (named, “phagicin”) is a product of phage replication; it is produced and can be detected before whole infective phage particles are released from bacterial cells. It could also be obtained by disruption of phage particles and is specific against HSV and vaccinia virus. Phagicin is sensitive to trypsin and pepsin but not deoxyribonuclease, ribonuclease and ultraviolet irradiation. Therefore, phagicin appears to be a phage protein interfering with the intracellular replication of viral DNA (Centifanto, 1968). Those data were confirmed by Meek et al., indicating that “phagicin” inhibits the synthesis of viral DNA but not the host DNA (Meek and Takahashi, 1968). In addition, Merril (1977) showed similar action for phage lambda. Those and other available data have been summarized (Międzybrodzki et al., 2005). Phage anti-viral action may be mediated via their nucleic acids as well as competition of phage and eukaryotic viruses for the same cellular receptors. Phage proteins have been shown to cause adjuvant-like action. Furthermore, phage may inhibit reactive oxygen species production relevant in the pathology of viral infections, Those phage-mediated effects may enhance anti-viral responses (Międzybrodzki et al., 2008; Górski et al., 2018b). This list does not exclude other possible mechanisms which remain largely unexplored, such as phage action at the level of natural killer (NK) cells. That indeed those findings may have some clinical significance and offer hope for their therapeutic potential is confirmed by our observations of increased protection against viral infections in patients who had completed PT (Weber-Dąbrowska et al., 2000). Correction of immunodeficiency with enhanced immunity to infections as a result of PT has also been reported by Russian authors (Lazareva et al., 2001). Moreover, a staphylococcal phage preparation was indicated for the treatment of viral warts, HSV types 1 and 2 and other viral conditions (Górski et al., 2009).

## Phage as a Potential Anti-Viral Agent

### Phage Downregulate NF-kappaB Activation

NF-kappa B transcription factors regulate the expression of genes involved in immune responses. To replicate and persist within their hosts, viruses have evolved strategies to exploit NF kappa B signaling for their benefit and avoid cellular mechanisms that eliminate the infection; in fact, its activation is a prerequisite for some viral infections (Nimmerjahn et al., 2004; Zhao et al., 2015; Struzik and Szulc-Dąbrowska, 2018). In contrast to HSV-1, T4 phage does not cause significant activation of NF-kappa B in human endothelial and epithelial cells. Moreover, preincubation of these cells with phage abolishes (endothelium) or significantly reduces (epithelium) NF kappa B activation (Górski et al., 2006). Interestingly, recently Zhang et al. (2018) confirmed and extended those findings by demonstrating that a staphylococcal phage can also abolish NF-kappa B activation by mechanisms unrelated to its anti-bacterial action. Those data may suggest that phage can inhibit NF kappa B activation triggered by different mechanisms. In our experiments phage interfered with HSV-induced mediator activation, whilst in those described by Zhang et al. (2018). Lipopolysaccharide (LPS) was used as an activator (Górski et al., 2006; Zhang et al., 2018). Further studies are necessary to determine whether the ability of phage to inhibit NF-kappa B activation depends on phage type and /or mechanisms of mediator activation.

The fact that a staph phage also inhibits activation of NF kappa B (Zhang et al., 2018) indicates that this is not a unique property of T4 phage. Although there are no data available on similar activities of purified phage proteins, some of those proteins have been demonstrated to mediate anti-inflammatory effects similar to functional phage (Miernikiewicz et al., 2016). Moreover, immobilized T4 phage show adhesive interactions with human T cells which can also be observed using purified gp24 phage protein (Górski et al., 2017). Nevertheless, currently available data are very scarce and further studies on the structure and immunobiological activities of phage proteins would shed more light on their potential application in therapy. Interestingly, in experiments reported by Van Belleghem et al. (2017) *Staphylococcus aureus* and *Pseudomonas aeruginosa* phage induced a highly comparable (although not identical) responses of the immune system. The authors hypothesize that modular nature of phage genomes and associated similar folds of phage capsid proteins may be responsible for those similarities. For example, gp23 and gp24 capsid proteins of *E. coli* phage T4 have a similar fold as that of the *E. coli* phage HK97 capsid protein. A single point mutation can change the serotype of the phage. The authors suggest that this might also be true for two *P. aeruginosa* phage that are homologous and only slightly differ in the induced immune response.

#### Lysogenic Conversion and Immune Response

Phage-dependentinhibition of NF-kappa B activity mentioned above was brought about by virulent phage. Furthermore, immunomodulatory effects reported by Van Belleghem et al. (2017) have also been mediated by such phage. On the other hand, phagicin production required induction of the lysogenic strain. No data are available on similar activities mediated by temperate phage. Interestingly, recent data from the Fischetti group have provided evidence that lysogeny plays a major role in the human adaptive immune response to bacterial infection. In those studies, prophages were shown to be responsible for a strong T and B cell immune response to lysogenic strains of bacteria (Sela et al., 2018). In contrast, lysogenic conversion has been shown to decrease phagocytosis of bacteria by phagocytes (Vaca-Pacheco et al., 1999; Secor et al., 2017). Thus, associations between lysogeny and immunity are complex and require further studies.

### Phage Inhibit Adsorption and Replication of Human Adenovirus and Modify the Expression of Genes Involved in Antimicrobial Immunity

In the first studies on the effect of phage on stages of infection by a pathogenic virus we found substantial dose-dependent inhibition by T4 phage of adsorption of HAdV to both A549 and HEK293 cell lines *in vitro* LPS was without effect. Moreover, T4 phage protected A549 cells from an HAdV-induced cytopathic effect (Międzybrodzki et al., 2013; Przybylski et al., 2015). These data suggest that T4 phage could be considered as a potential novel antiviral agent. The capacity of the phage to inhibit HAdV infection at the stage of viral replication suggests that phage could also interfere with viruses using cellular receptors other than those used by HAdV. Notably, the anti-viral effect of phage was measured as the decrease of the end-point HAdV infectious titer. Further *in vitro* studies were carried out on the effects of T4 and the staphylococcal phage A5/80 on HAdV DNA synthesis (real-time PCR) and the expression of its early and late genes (measured at the level of mRNA synthesis) in an A549 cell culture. Continuous incubation of adenovirus-infected cells with T4 phage significantly reduced the level of adenoviral DNA synthesis (this effect was not observed with the staphylococcal phage). Coincubation with a high titer of T4 phage was required for inhibition of HAdV early gene expression. Late adenoviral gene expression was reduced by preincubation (application of phage prior to HAdV infection) and coincubation with both phage when a low HAdV titer was used. In contrast, when a high HAdV titer was applied, both incubation and preincubation with T4 phage were inhibitory whilst a staphylococcal phage was inhibitory only when continuous incubation with cells was applied. Those results suggest that the inhibitory effect of phage on HAdV infection may differ and may be partially explained by their influence on the expression of early and late adenoviral genes (Przybylski et al., 2018). When the effect of both phage on the expression of genes involved in antimicrobial immunity by the A549 cell line from human lung was studied, the most striking phenomenon was marked (>10-fold) enhancement of a gene coding for interleukin-2 (Il-2) by a staphylococcal phage (Borysowski et al., 2018). This effect is of particular interest in the context of the well-known role of NK cells in the immune response to viruses (Hammer et al., 2018) and the ability of Il-2 to induce those cells – even in ultra-low doses (Fehniger et al., 2000; Ito et al., 2014). Interestingly, a resident NK cell population is present in the human lung and may provide early and important control of viral infection (Cooper et al., 2018). Moreover, NK cells also exhibit activity against a variety of bacteria, e.g., by secretion of the soluble molecules perforin and granulysin. Mice infected with *Shigella* and lacking B and T cells but with normal NK cells have higher survival rates and lower bacterial titers than mice which lack all three cell types. This suggests that phage-induced Il-2 dependent activation of NK-mediated antimicrobial activity may contribute to beneficial effects of PT, especially during prolonged phage administration cumulative median duration of successful phage therapy is 43 days (Międzybrodzki et al., 2012). NK cells could be a promising agent in antimicrobial immunotherapy, as data strongly suggest that these cells are active against not only viral but also bacterial and fungal pathogens (Schmidt et al., 2018).

## Phage as a Potential Anti-Fungal Agent

### Filamentous Phage and Inhibition of Fungal Metabolism

There are some suggestions that bacteriophage may adversely impact fungal growth. These studies involve a bacteriophage produced by *P. aeruginosa* (*Pa*), a Gram negative bacteria, and the fungal pathogens *A. fumigatus* (*Af*) and *Candida albicans* (*Ca*).

*Pa* is known to have complex and clinically relevant interactions with *Af*, most notably in lung infections in patients with Cystic Fibrosis (CF). *Pa* (Govan and Deretic, 1996; Bjarnsholt et al., 2009; Høiby et al., 2010) and *Af* (Amin et al., 2010; Baxter et al., 2013; Kaur and Singh, 2014) are among the most common bacterium and fungus infecting airways in CF, respectively. Both organisms are associated with more rapid declines in CF pulmonary function (Schønheyder et al., 1985; Nicolai et al., 1990; Shoseyov et al., 2006; Fillaux et al., 2012; Speirs et al., 2012; Ramsey et al., 2014) and can cause invasive disease (Yeldandi et al., 1995; Cahill et al., 1997; Nunley et al., 1998) as well as other complications (Stevens et al., 2003; Botha et al., 2008; Garantziotis and Palmer, 2009). Co-colonization of the lungs of CF patients with *Pa* and *Af* can result in more severe pulmonary disease. However, *Pa* can be inhibitory to *Af* through the production of multiple antimicrobials such as pyocyanin (5-N-methyl-1-hydroxyphenazine) (Mangan, 1969; Blyth, 1971; Kerr et al., 1999; Briard et al., 2015), 1-hydroxyphenazine (Mangan, 1969; Kerr et al., 1999; Briard et al., 2015), and phenazine-1-carboxamide and phenazine-1-carboxylic acid (Briard et al., 2015). This inhibition is most closely associated with small colony variants of Pa although there is considerable heterogeneity between isolates (Anand et al., 2018).

Recently, it was reported that these interactions between *Pa* and *Af* are mediated in part by Pf, a filamentous bacteriophage in the genus Inovirus. Pf is prevalent amongst clinical *Pa* isolates, (Rakonjac et al., 2011) including epidemic strains (Knezevic et al., 2015). Pf exists as a prophage within *Pa* and is produced in large quantities within *Pa* biofilms (Rice et al., 2008; Secor et al., 2015).

In a series of elegant studies led by Dr. David Stevens at Stanford, it was reported that Pf phage *Pa* inhibits *Af* biofilms. Pf phage produced by *Pa* binds iron and can sequester this critical resource in ways that deny it to *Af*, thereby restricting fungal growth (Penner et al., 2016). Iron concentrations are known to impact the severity of CF lung infections (Reid et al., 2007) and Fe^3+^ is a critical resource for *Af* (Nazik et al., 2015). We reported that supplemental iron could overcome Pf4-mediated inhibition of *Af* metabolism and we demonstrated that Pf4 binds iron (Penner et al., 2016). This finding was consistent with previous reports that supplemental iron also overcame the inhibition seen with *Pa* supernatants (Stevens et al., 2003). It was also consistent with other work suggesting that *Af* is exquisitely sensitive to the concentration of iron in the surrounding milieu (Nazik et al., 2015). A similar inhibitory effect of Pf4 phage was observed on the *in vitro* growth of another fungal species, *Candida albicans* (Nazik et al., 2017).

Interestingly, the ability of Pf phage to sequester iron and limit the growth of Af was limited to some phage strains and not others. Inhibition of *Af* metabolism was seen with Pf4 from *Pa* strain PA01 but not with other filamentous phage tested, including fd, from *E. coli*, and Pf1, and from *Pa* strain PAK. Consistent with this difference, Pf4 was found to bind iron more efficiently than Pf1. Some of this Pf4-Pf1 difference may perhaps reflect the greater length and proportionately increased charge area of Pf4 (Rakonjac et al., 2011). This ability to sequester iron may therefore be specific to only some filamentous phage and the broader relevance of these findings is therefore unknown.

However, there are additional levels of complexity to these interactions. In subsequent studies, Stevens and his colleagues reported that pyoverdine likewise chelates iron and denies this resource to *Af* (Secor et al., 2017; Sass et al., 2018). However, the relative importance of these mechanisms and the impact of Pf levels on these effects are unknown. Moreover, *Pa* are known to produce multiple other molecules that are inhibitory to *Af* (Mangan, 1969; Blyth, 1971; Kerr et al., 1999; Briard et al., 2015).

Together these results highlight the potential impact and substantial uncertainties inherent in using bacteriophage to influence fungal communities colonizing sites of chronic infection.

## Conclusion

The data presented in this article are still preliminary. Nevertheless, they suggest novel avenues for further research on phage immunobiology. It is rather evident that in the face of growing antibiotic resistance anti-bacterial action of phage offers the best perspectives for their therapeutic application. At any rate, it cannot be excluded that – according to the drug repurposing strategy – there may exist prospects for phage application unrelated to their anti-bacterial action. Further studies are necessary to determine whether PT may also bring some benefits in viral and fungal infections.

## Author Contributions

AG and PB drafted the main parts of the manuscript. MP, JB, RM, EJ-M, and BW-D contributed to parts of the manuscript. All authors approved the manuscript.

## Conflict of Interest Statement

AG, BW-D, JB, and RM are co-inventors of patents owned by the Institute and covering phage preparations. The remaining authors declare that the research was conducted in the absence of any commercial or financial relationships that could be construed as a potential conflict of interest.
